# The Importance of Considering Personal Recovery for Eating Disorders

**DOI:** 10.1002/erv.70116

**Published:** 2026-04-21

**Authors:** Andrew Allen, Maddison Tunstall, Catherine Houlihan, Kay Pozzebon, Daniel B. Fassnacht, Kathina Ali

**Affiliations:** ^1^ School of Health University of the Sunshine Coast Sippy Downs Queensland Australia

**Keywords:** clinical recovery, clinical remission, eating disorders, personal recovery, sexual identity

## Abstract

**Objective:**

Definitions of recovery from eating disorders (EDs) have traditionally emphasised symptom reduction and functional restoration. However, growing research highlights the importance of integrating personal recovery, defined by self‐acceptance, autonomy, and psychological wellbeing. This study explored the relationship between partial clinical remission and personal recovery in individuals with lived experience of an ED.

**Method:**

A total of 234 participants (*N* = 234; *M*_age_ = 28.10; 89.3% female; 49.6% non‐heterosexual) were recruited online and completed measures assessing ED symptom severity, quality of life, and personal recovery. Criteria for partial clinical remission were based on established cut‐offs for measures of ED symptomology. Personal recovery was defined by self‐endorsement of a validated recovery framework.

**Results:**

Chi‐square tests revealed that rates of personal recovery (52.1%) were significantly higher than rates of partial clinical remission (22.6%). While clinical remission increased the likelihood of personal recovery (OR = 6.46), many participants endorsed personal recovery despite not meeting clinical criteria. No significant differences in personal recovery were found between ED types or between heterosexual and non‐heterosexual participants as an exploratory analysis.

**Conclusions:**

These findings underscore the importance of incorporating personal recovery constructs into ED assessment, treatment, and outcome monitoring.

## Highlights

- Personal recovery often extends beyond symptom remission, revealing a gap between clinical and lived recovery.
- Recovery experiences appear similar across eating disorder types and sexual identity.
- Integrating personal recovery concepts can enrich treatment and support lasting change.

## 1 Introduction

Feeding and eating disorders (EDs) are characterised by disturbed eating or eating‐related behaviours that impair psychosocial functioning (American Psychiatric Association 2022). Common EDs include anorexia nervosa (AN), bulimia nervosa (BN), binge eating disorder (BED), and other specified feeding or eating disorder (OSFED; Devoe et al. 2023; Tannous et al. 2022). Globally, the prevalence of EDs has increased, with a point prevalence increase from 3.5% to 7.8% between 2000 and 2018 (Galmiche et al. 2019). More recently, a trend analysis showed an approximate 43% increase in prevalence, incidence, and disease burden among adolescents and young adults from 1990 to 2021 (Liu et al. 2025). In Australia, an estimated 16.3% of the population is affected by eating disorders or disordered eating (Hay et al. 2015), with recent global estimates indicating that Australia has the highest age‐standardised prevalence and burden of eating disorders worldwide (Liu et al. 2025). EDs can occur from childhood to older adulthood (Christian et al. 2020; Morris et al. 2022), with heightened risk among cisgender females and sexually or gender diverse individuals (Brown et al. 2020; Himmerich et al. 2024; Riddle et al. 2024; White et al. 2024). Despite growing awareness, EDs continue to impose substantial individual (e.g., low self‐worth, emotional distress), social (e.g., stigma, isolation), physical (e.g., obesity, emaciation), and financial burdens (Bianchi et al. 2020; Bray et al. 2022; St‐Pierre et al. 2023; Tannous et al. 2022), costing billions (USD) each year (Streatfeild et al. 2021).

While existing treatment approaches for EDs have demonstrated efficacy, treatment‐seeking is low (Ali et al. 2025), recovery rates remain variable, and relapse is common (Lydecker et al. 2019; Miskovic‐Wheatley et al. 2023; Mountford et al. 2021). The lack of consensus regarding the definition and assessment of ED recovery may complicate such variability (de Rijk et al. 2024; Huryk et al. 2024). Traditionally, recovery has been conceptualised predominantly through a clinical lens (e.g., clinical remission), focusing on objective markers such as symptom reduction, weight restoration, and healthy eating behaviours (Khalsa et al. 2017; Wade and Lock 2020). Yet, such criteria may be misaligned with the lived experiences of individuals with EDs (Fogarty and Ramjan 2016; Kinnaird and Cooper 2024; Rance et al. 2017) or overlook aspects of recovery that are personally significant to the individual (Ashworth et al. 2009; de Vos et al. 2017). Accordingly, there is increasing recognition of ‘personal recovery’ regarding EDs, emphasising individual growth, autonomy, emotional wellbeing, and reconnection with identity and relationships, even in the presence of ongoing symptoms (Leamy et al. 2011; Piot et al. 2020; Wetzler et al. 2020). This view is congruent with recent research demonstrating that individuals can experience high levels of mental wellbeing, despite the existence of eating disorder symptoms (Scutt et al. 2023, 2025). Frameworks such as CHIME (Connectedness, Hope, Identity, Meaning, Empowerment) have been adapted to the ED field to capture these broader experiences (Piot et al. 2020; Wetzler et al. 2020).

Although limited, literature on EDs has found that the CHIME framework supports personal recovery. Wetzler et al. (2020) conducted a systematic review and qualitative meta‐synthesis of 20 studies on those recovered from an ED to identify themes of personal recovery, which were compared to the CHIME framework. Findings suggested that aspects of the CHIME model were closely associated with personal recovery within EDs. Similarly, Piot et al. (2020) interviewed young adults who experienced severe AN during adolescence to understand personal recovery. Seven themes were identified. The authors specifically highlighted that their findings built on the CHIME model by noting the importance of group identity (Connectedness), recovery over time (Hope), dealing with stigma (Identity), connection between mind and body (Meaning), and being vulnerable and working on oneself (Empowerment; Piot et al. 2020). These features of personal recovery can be protective against ED relapse (McCabe et al. 2019). Therefore, personal recovery, as defined by models like CHIME, may help identify aspects of ED recovery beyond improvements in clinical symptomatology, which in turn may support the recovery process.

Indeed, emerging evidence suggests that incorporating personal recovery principles into ED treatment may improve long‐term outcomes, quality of life, and relapse prevention. In a prospective study, McNamara et al. (2022) found that fostering connectedness and identity reconstruction was associated with reduced symptom severity and improved psychological wellbeing in people with EDs. Similarly, interventions that promote self‐acceptance, autonomy, and meaning making have been linked to enhanced motivation for change and sustained engagement in treatment (Cornelissen and Tovée 2021; Piot et al. 2020). Qualitative research further indicates that recovery plans co‐designed with clients, incorporating personal recovery goals alongside symptom reduction targets, are perceived as more relevant and empowering, leading to greater adherence and reduced relapse risk (Hurst et al. 2022; Wetzler et al. 2020). Collectively, these findings support the integration of personal recovery approaches with traditional clinical targets to foster more durable and person‐centred recovery in EDs.

### 1.1 Current Study

Despite increasing calls to incorporate both clinical and personal dimensions of recovery (de Vos et al. 2018; Wetzler et al. 2020), the literature continues to disproportionately emphasise symptom‐focused conceptualisations. While personal recovery frameworks may offer promising insights, empirical research examining how these models intersect with clinical remission remains limited. Greater clarity is needed around the degree to which these dimensions align or diverge, and how this relationship may differ across ED subtypes.

The current study addresses this gap by comparing rates of partial clinical remission and personal recovery among individuals with lived or living experience of an ED. Using validated measures of ED psychopathology and impairment, alongside participant endorsement of personal recovery, the study aimed to clarify whether clinical and personal recovery represent overlapping or distinct constructs. A secondary objective was to examine whether personal recovery differs between ED types.

## 2 Method

### 2.1 Procedure and Participants

Ethics approval was obtained from a University Human Research Ethics Committee. Participants were recruited through Facebook advertisements, ED recovery‐related groups, snowball sampling on social media (e.g., Facebook, Instagram), and posters distributed across universities and local businesses in Australia. Third‐party ED organisations (e.g., the Butterfly Foundation, Australia and New Zealand Academy for Eating Disorders) also promoted the study. Advertisements included ‘Do you have a lived or living experience of an eating disorder?’ We want to hear from you about your recovery journey. Participants accessed the survey via a link or QR code and were presented with an information sheet before providing consent. The survey included demographic items and measures. Attention check items (e.g., ‘What is 16 + 10?’) were embedded to ensure valid responses; data from participants who failed these checks were excluded. Upon completion, participants were thanked for their time and provided with contact information for relevant support services (e.g., Butterfly Foundation, Lifeline, Beyond Blue).

Participants aged ≥ 18 years with lived or living experience of an ED were recruited across Australia. A sample size of *N* = 122 was recommended to detect a medium effect with 80% power (Faul et al. 2007). In total, 234 participants were included in the study (*M*_age_ = 28.10, SD = 9.34). Most identified as female (89.3%) and White/European (87.2%), with 49.6% identifying as non‐heterosexual. Over half (51.3%) reported a current ED diagnosis, 42.3% a past diagnosis, and the remainder reported undiagnosed or relapsing ED experiences. Full demographic details are provided in Table 1.

**TABLE 1:** Demographics of survey participants (*N* = 234).

	*n*	%
Gender		
Male	16	6.8
Female	209	89.3
Other self‐described (e.g., agender, non‐binary, genderqueer, gender non‐conforming)	8	3.4
Prefer not to answer	1	0.4
Australian and/or TSI origin		
Aboriginal	13	5.6
TSI	5	2.1
Aboriginal and TSI	3	1.3
Neither Aboriginal/TSI	213	91.0
Ethnicity		
White/European	204	87.2
Asian/Indian	16	6.8
African	2	0.9
Other	12	5.1
Sexual identity		
Heterosexual	118	50.4
Gay or lesbian	23	9.8
Bisexual	55	23.5
Pansexual	4	1.7
Asexual	17	7.3
Queer	9	3.8
Something else (aro/ace spectrum)	18	7.7
Prefer not to say/unsure	8	3.4
Highest education level		
Tertiary	175	74.9
Secondary	59	25.2
Employment		
Full‐time	57	24.4
Part‐time	56	23.9
Casual	66	28.2
Unemployed	48	20.5
Other	7	3
University student		
Yes	114	48.7
No	120	51.3
ED diagnosis		
Currently diagnosed	120	51.3
Previous diagnosis	99	42.3
Might have ED, not diagnosed	6	2.6
Might have had an ED in past, not diagnosed	3	1.3
Other	6	2.6

*Note:* Participants could select more than one sexual identity category; therefore, totals exceed the sample size.

Abbreviations: ED, eating disorder; TSI, Torres Strait Islander.

### 2.2 Materials

#### 2.2.1 Demographics

Demographic information, including age, gender, education, employment status, and university enrolment, was collected. Sexual identity was assessed via self‐report using categorical options (e.g., heterosexual, bisexual, gay, lesbian, pansexual, queer, asexual). Gender identity and intersex status were not assessed.

#### 2.2.2 Eating Disorder Treatment History

Sixteen items assessed participants' experiences with ED treatment, including treatment type (e.g., ‘What type of eating disorder treatment did you receive?’) and perceived helpfulness (e.g., ‘Overall, how helpful was the eating disorder treatment you received?’). These items were adapted from the Eating Disorder Treatment and Management survey (McLean and Fuller‐Tyszkiewicz 2024) and reviewed by a panel of researchers and clinicians with expertise in EDs.

#### 2.2.3 Eating Disorder Examination Questionnaire (EDE‐Q)

The EDE‐Q (Fairburn and Beglin 1994) is a 28‐item self‐report measure used to assess ED symptom severity and determine clinical remission status. Items are rated on a 6‐point scale based on symptom frequency over the past 28 days. The measure includes subscales for dietary restraint, eating concern, shape concern, and weight concern, which contribute to a global severity score (Rand‐Giovannetti et al. 2020). The EDE‐Q has demonstrated strong reliability and validity for online administration (Jenkins and Rienecke 2022), with acceptable to high internal consistency in this study (total score *α* = 0.97; Restraint *α* = 0.91; Eating Concern *α* = 0.82; Shape Concern *α* = 0.93; Weight Concern *α* = 0.86). The EDE‐Q has also demonstrated acceptable psychometric performance in some sexual and gender minority samples (Nagata et al. 2023; de Oliveira Júnior et al. 2023).

#### 2.2.4 Clinical Impairment Assessment Questionnaire (CIA)

The CIA (Bohn and Fairburn 2008) is a 16‐item self‐report scale measuring impairment from ED symptoms over the past 28 days. Items are rated on a 4‐point Likert scale and cover personal, social, and cognitive impairment domains. A total score (range 0–48) indicates overall ED‐related impairment. The CIA has demonstrated strong validity and reliability (Maraldo et al. 2021), with excellent internal consistency in the current study (*α* = 0.97). The CIA was used to identify impairment level and categorise participants as either within clinical remission or partial clinical remission.

#### 2.2.5 Personal Recovery

Participants were presented with the following definition of personal recovery: Recovery of an eating disorder includes self‐acceptance, positive relationships, personal growth, and a decrease in eating disorder behaviour/cognitions, self‐adaptability/resilience and autonomy (de Vos et al. 2017). Participants rated the extent to which they related to the definition using a visual analogue scale from 1 (*strongly disagree*) to 5 (*strongly agree*).

### 2.3 Data Analysis

Data were analysed using SPSS 29.0. Descriptive statistics were computed for demographics. Chi‐square tests were used to compare rates of personal recovery and partial clinical remission, and personal recovery across ED types. Following prior research (Jenkins et al. 2019; Kinnaird and Cooper 2024; Wade and Lock 2020), partial clinical remission was defined as meeting all of the following: EDE‐Q Global ≤ 2.53, Restraint ≤ 2.57 (based on Fairburn and Beglin 1994), and CIA score ≤ 16. Partial clinical remission was used to reflect clinically meaningful symptom improvement short of full remission (i.e., sustained symptom absence over time). Personal recovery was defined as a rating of ≥ 4 (on a 1–5 scale) in response to de Vos et al. (2017, 2018) recovery definition. ED types were grouped into four categories: AN/atypical AN, BN/subthreshold BN, BED/subthreshold BED, and other ED/ED behaviours. An exploratory analysis also examined personal recovery across sexual identity groups (heterosexual vs. non‐heterosexual), given the high representation of non‐heterosexual participants. Participants who selected both a heterosexual and a non‐heterosexual identity (*n* = 3) were classified as non‐heterosexual. Participants who selected options indicating uncertainty or preference not to disclose (*n* = 8) were excluded from the sexual identity analysis, resulting in an analytic sample of *N* = 226.

## 3 Results

### 3.1 Data Cleaning and Assumptions Testing

Participants were included if they provided informed consent, passed attention checks, and completed the key survey measures. A total of 32 incomplete or invalid responses were excluded, resulting in a final sample of 234 participants. Assumptions for chi‐square tests were met, including independence of observations and adequate expected cell counts (≥ 5).

### 3.2 ED Characteristics

Most participants self‐reported a past or current diagnosis of AN (*n* = 132, 56.4%) or atypical AN (*n* = 28, 12.0%). Others reported BN (*n* = 19, 8.1%), BED (*n* = 18, 7.7%), or OSFED or ‘disordered eating’ (*n* = 12, 5.1%). Additional diagnoses included avoidant/restrictive food intake disorder (ARFID; *n* = 11, 4.7%), night eating syndrome (*n* = 5, 2.1%), unsure (*n* = 4, 1.7%), purging (*n* = 3, 1.3%), subthreshold BN (*n* = 1, 0.4%), and subthreshold BED (*n* = 1, 0.4%). See Table 2 for full ED characteristics and EDE‐Q/CIA descriptive statistics.

**TABLE 2:** ED and ED questionnaire characteristics.

	*n*	%	*M*	SD	Range
ED type					
AN	132	56.4			
BN	19	8.1			
BED	18	7.7			
Atypical AN	28	12.0			
Subthreshold BN	1	0.4			
Subthreshold BED	1	0.4			
Purging	3	1.3			
Night eating	5	2.1			
ARFID	11	4.7			
Unsure	4	1.7			
Other	12	5.1			
EDE‐Q					
Restraint	234	100	2.57	2.06	0–6
Eating concern	234	100	2.41	1.69	0–6
Shape concern	234	100	3.69	1.84	0–6
Weight concern	233	99.6	3.48	1.81	0–6
Global score	233	99.6	3.03	1.72	0–6
CIA					
Global score	234	100	21.47	14.84	0–48

Abbreviations: AN, anorexia nervosa; ARFID, avoidant/restrictive food intake disorder; BED, binge eating disorder; BN, bulimia nervosa; CIA, Clinical Impairment Assessment; ED, eating disorder; EDE‐Q, Eating Disorder Examination Questionnaire.

### 3.3 Rates of Personal Recovery Versus Partial Clinical Remission

To test whether rates of partial clinical remission differed from rates of personal recovery, a chi‐square test was conducted using these two categorical variables. Most participants (*n* = 181, 77.4%) did not meet the criteria for partial clinical remission, with 44.0% of this group also not meeting the criteria for personal recovery. Overall, 52.1% (*n* = 122) met the personal recovery criteria, compared to 22.6% (*n* = 53) who met the criteria for partial clinical remission. This difference was statistically significant, *χ* ^2^(1, *N* = 234) = 26.19, *p* < 0.001. Participants in partial clinical remission were 6.46 times more likely to report personal recovery. However, 63.9% of those who self‐identified as personally recovered did not meet criteria for partial clinical remission. Table 3 shows the distribution and percentages of participants within the categories for personal recovery and partial clinical remission.

**TABLE 3:** Contingency table of observed frequencies of partial clinical remission and personal recovery.

		Personal recovery		Total
		No	Yes	
Partial clinical remission	Yes	9	44	53 (22.6%)
	No	103	78	181 (77.4%)
	Total	112 (47.9%)	122 (52.1%)	*N* = 234

### 3.4 Personal Recovery Across Types of EDs

To test whether rates of personal recovery differed by ED type, a chi‐square test was conducted using personal recovery status and ED type as categorical variables. Participants with AN or atypical AN (*n* = 160) had the lowest rate of personal recovery (49.4%), while recovery rates were higher among those with other EDs or related behaviours (57.1%), BED/subthreshold BED (57.9%), and BN/subthreshold BN (60%). Overall, 52.1% of the sample met personal recovery criteria. However, differences across ED types were not statistically significant, *χ* ^2^(3, *N* = 234) = 1.59, *p* = 0.662. Table 4 shows the distribution and percentages of participants within the categories for personal recovery and ED type.

**TABLE 4:** Contingency table of observed frequencies of personal recovery and ED type.

		Personal recovery		Total
		No	Yes	
ED type	AN/atypical AN	81	79	160 (68.4%)
	BN/subthreshold BN	8	12	20 (8.5%)
	BED/subthreshold BED	8	11	19 (8.1%)
	Other EDs/ED behaviour	15	20	35 (15.0%)
	Total	112 (47.9%)	122 (52.1%)	*N* = 234

Abbreviations: AN, anorexia nervosa; BED, binge eating disorder; BN, bulimia nervosa; ED, eating disorder.

### 3.5 Exploratory Analysis: Rates of Personal Recovery and Sexual Identity

Due to the sample containing a high number of non‐heterosexual participants, a third, exploratory analysis was conducted. An exploratory chi‐square test examined whether personal recovery rates differed by sexual identity. Recovery rates were similar between participants identifying as heterosexual (53.9%) and those identifying as non‐heterosexual (50.5%). As shown in Table 5, this difference was not statistically significant, *χ* ^2^(1, *N* = 226) = 0.27, *p* = 0.602.

**TABLE 5:** Contingency table of observed frequencies of personal recovery and sexual identity.

		Personal recovery		Total
		No	Yes	
Sexual identity	Heterosexual	53	62	115 (50.9%)
	Non‐heterosexual	55	56	111 (49.1%)
	Total	108 (47.8%)	118 (52.2%)	*N* = 226

*Note:* Not all participants completed items on sexual identity.

## 4 Discussion

The study aimed to explore rates of personal recovery and partial clinical remission, as well as rates of personal recovery between EDs. Given the high rates of participants who identified as non‐heterosexual, the potential relationship between rates of personal recovery and sexual identity was also examined.

### 4.1 Rates of Personal Recovery Are Higher Than Rates of Partial Clinical Remission

The findings demonstrate that self‐reported rates of personal recovery were significantly higher than rates of partial clinical remission among individuals with lived ED experience. Over half the sample (52.1%) met criteria for personal recovery, compared with 22.6% who met clinical remission. These results align with prior research (Kinnaird and Cooper 2024) and support calls to incorporate personal recovery into ED recovery frameworks (Costin and Grabb 2011; de Vos et al. 2017, 2018; Wetzler et al. 2020). Although clinical remission increased the likelihood of personal recovery, 63.9% of those who identified as personally recovered did not meet clinical criteria, highlighting a possible disconnect between symptom‐based and person‐centred definitions. This gap suggests that conventional clinical metrics may lack relevance for many, as they overlook key personal recovery factors such as self‐acceptance, autonomy, and psychological growth (de Vos et al. 2017, 2018). The observed relationship between clinical and personal recovery is consistent with previous findings (Kinnaird and Cooper 2024), reinforcing the notion that both dimensions are important when conceptualising recovery.

### 4.2 Rates of Personal Recovery Are Independent of ED Type

Personal recovery rates did not significantly differ between ED types. Although individuals with AN or atypical AN reported the lowest rates of personal recovery, this difference was not statistically significant. One possible explanation is the ego‐syntonic nature of AN, whereby symptoms such as extreme dietary restriction and pursuit of thinness are often experienced as congruent with the individual's values or identity (Asaria 2023; Di Lodovico et al. 2024), potentially creating additional barriers to the self‐acceptance and identity transformation central to personal recovery. This interpretation aligns with theoretical models (e.g., de Vos et al. 2017, 2018) that highlight autonomy and identity reconstruction as essential to personal recovery. Additionally, overrepresentation of AN in the sample, coupled with smaller subsamples for other ED types, may have limited statistical power to detect true between‐group differences. Future studies with more diagnostically balanced samples are needed to explore whether certain ED profiles pose greater challenges for achieving personal recovery.

### 4.3 Rates of Personal Recovery Are Comparable Across Sexual Identity

An exploratory analysis found no significant differences in personal recovery rates between heterosexual and non‐heterosexual participants, suggesting that a dichotomous assessment of sexual identity was not associated with endorsement of personal recovery. While higher rates of ED risk and severity are noted among sexually and gender diverse populations (Himmerich et al. 2024), the present findings suggest that this may not necessarily extend to personal recovery outcomes. One possible explanation is that resilience and community support within sexual minority populations may buffer recovery pathways, offsetting initial vulnerabilities (White et al. 2024). Alternatively, recovery processes may be more strongly influenced by intrapersonal factors, such as self‐acceptance and psychological growth, than by sexual identity exclusively. Notably, most research in this area has focused on ED onset and risk (Castellini et al. 2020; Himmerich et al. 2024; O’Flynn et al. 2023; White et al. 2024), rather than recovery trajectories. As such, this study highlights the possibility for further research to explore how recovery is experienced across diverse sexual and gender identities. Future work would benefit from disaggregating sexual minority subgroups and considering intersecting variables such as minority stress, access to affirming care, and body image ideals within specific communities.

### 4.4 Strengths and Implications

This study contributes to the growing literature advocating for a broader definition of ED recovery. Findings support integrating personal recovery frameworks, such as de Vos et al. (2017), into clinical assessment and intervention. Doing so could help tailor treatment goals to individuals' lived experiences, strengthen relapse prevention, and improve long‐term intervention outcomes. For individuals with AN, interventions that promote self‐acceptance and challenge identity fusion with the disorder may be particularly beneficial. Moreover, the inclusion of non‐heterosexual participants offers an important step towards addressing recovery within underserved groups.

### 4.5 Limitations and Future Directions

Several limitations warrant consideration. The cross‐sectional, quantitative design limits causal interpretations and depth of understanding. Gender was assessed using a single‐item measure, and sex assigned at birth was not collected; therefore, transgender status could not be reliably classified, and gender diversity may be underrepresented. The broad categorisation of sexual identity may have obscured meaningful differences between subgroups. Personal recovery was assessed using a single‐item endorsement, which may not fully capture the multidimensional nature of personal recovery described in prior literature. Ethnicity categories were broad and may have conflated race, ancestry, and geographic origin. Further, reliance on self‐report measures introduces potential bias, and there was an overrepresentation of participants with AN, limiting generalisability to other EDs. Future research would benefit from: (1) adopting longitudinal studies to understand nuanced components of personal recovery for those with an ED; (2) measuring different dimensions of personal recovery in the ED context; (3) recruiting more diverse and diagnostically balanced samples; and (4) exploring subgroup‐specific experiences within sexual minority communities.

## 5 Conclusion

This study highlights the disconnect between clinical and personal definitions of ED recovery. Personal recovery was more commonly endorsed than partial clinical remission, and not significantly influenced by ED type or sexual identity. These findings underscore the importance of expanding recovery frameworks beyond symptom reduction to include psychological wellbeing, identity, and meaning‐making. Incorporating personal recovery into research, assessment, and intervention may enhance treatment relevance and promote more sustainable outcomes for individuals with EDs.

## Conflicts of Interest

The authors declare no conflicts of interest.

## Data Availability

The data that support the findings of this study are available from the corresponding author upon reasonable request.
